# Supplementary material for: Aromatase inhibition remodels the clonal architecture of estrogen-receptor-positive breast cancers
Source: Nat Commun. 2016 Aug 9;7:12498. doi: 10.1038/ncomms12498 (PMC4980485; doi:10.1038/ncomms12498)
Supplement: Supplementary Information — Supplementary Figures 1-8, Supplementary Notes 1-5, Supplementary Data 1-12, and Supplementary Software 1-2. [file ncomms12498-s1.pdf]

Supplementary Figures

Supplementary Figure 1: Somatic copy number alterations detected by CopyCat and displayed after purity correction.

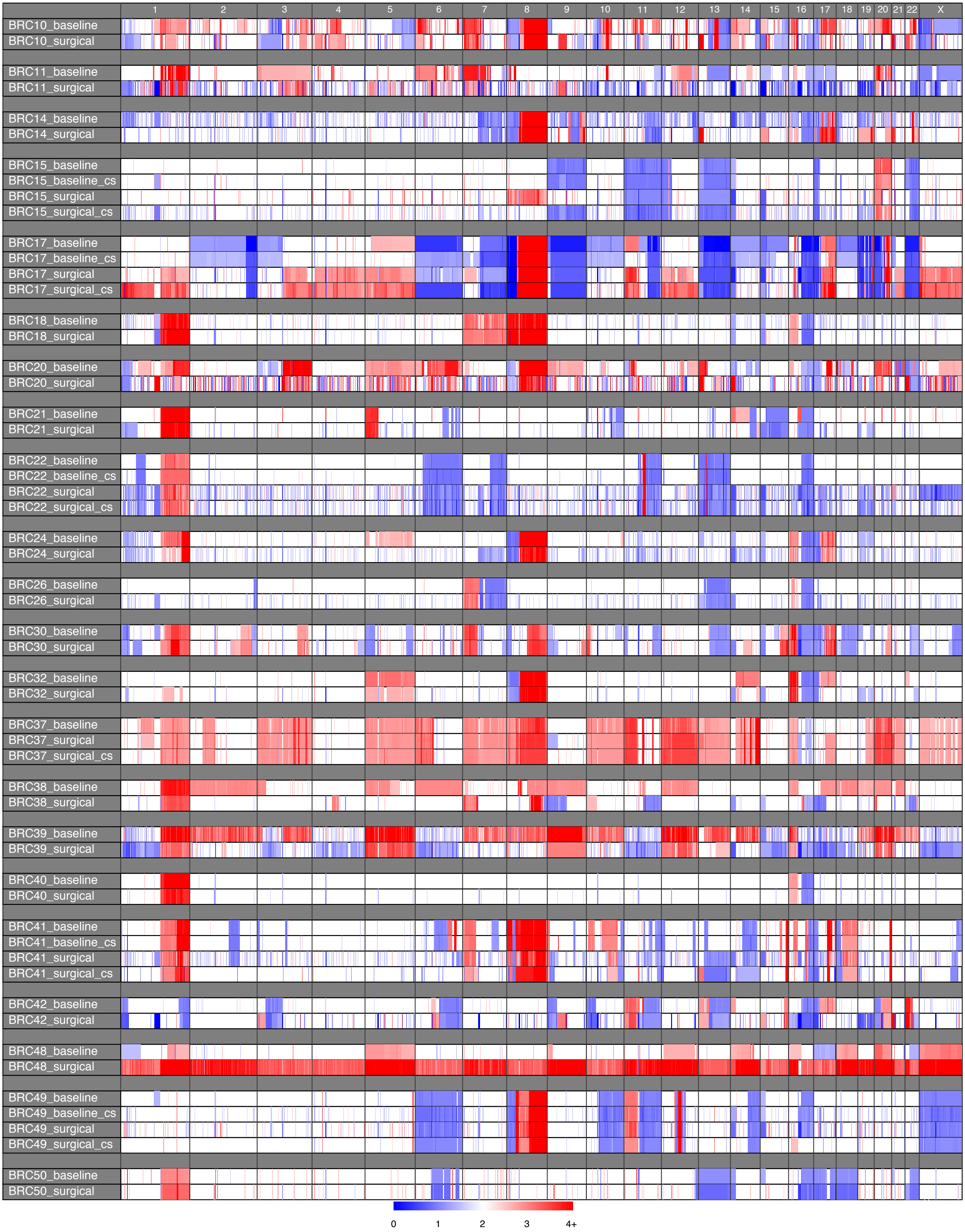

1 2 3 4 5 6 7 8 9 10 11 12 13 14 15 16 17 18 19 20 21 22 X
BRC10_baseline
BRC10_surgical
BRC11_baseline
BRC11_surgical
BRC14_baseline
BRC14_surgical
BRC15_baseline
BRC15_baseline_cs
BRC15_surgical
BRC15_surgical_cs
BRC17_baseline
BRC17_baseline_cs
BRC17_surgical
BRC17_surgical_cs
BRC18_baseline
BRC18_surgical
BRC20_baseline
BRC20_surgical
BRC21_baseline
BRC21_surgical
BRC22_baseline
BRC22_baseline_cs
BRC22_surgical
BRC22_surgical_cs
BRC24_baseline
BRC24_surgical
BRC26_baseline
BRC26_surgical
BRC30_baseline
BRC30_surgical
BRC32_baseline
BRC32_surgical
BRC37_baseline
BRC37_surgical
BRC37_surgical_cs
BRC38_baseline
BRC38_surgical
BRC39_baseline
BRC39_surgical
BRC40_baseline
BRC40_surgical
BRC41_baseline
BRC41_baseline_cs
BRC41_surgical
BRC41_surgical_cs
BRC42_baseline
BRC42_surgical
BRC48_baseline
BRC48_surgical
BRC49_baseline
BRC49_baseline_cs
BRC49_surgical
BRC49_surgical_cs
BRC50_baseline
BRC50_surgical
0 1 2 3 4+

Supplementary Figure 2: Expression Clustering of all samples. Heatmap display of hierachical clustering of the top 5000 most variably-expressed genes in all samples

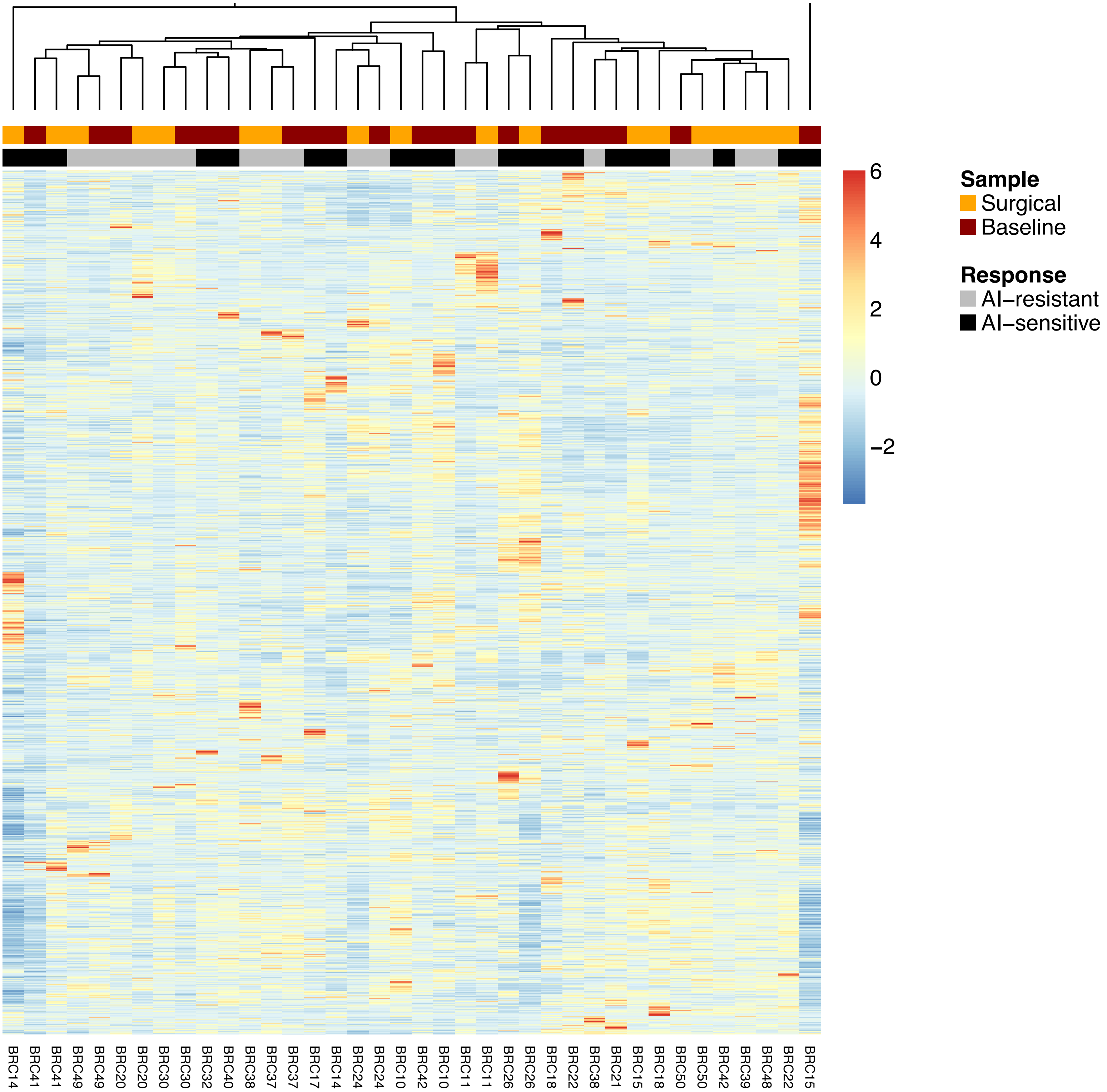
6
4
2
0
−2
Sample
Surgical
Baseline
Response
AI−resistant
AI−sensitive
BRC14
BRC41
BRC41
BRC49
BRC49
BRC20
BRC20
BRC30
BRC30
BRC32
BRC40
BRC38
BRC37
BRC37
BRC17
BRC14
BRC24
BRC24
BRC10
BRC42
BRC10
BRC11
BRC11
BRC26
BRC26
BRC18
BRC22
BRC38
BRC21
BRC15
BRC18
BRC50
BRC50
BRC42
BRC39
BRC48
BRC22
BRC15

Supplementary Figure 3: Expression Clustering of baseline samples. Heatmap display of hierachical clustering of the top 5000 most variably-expressed genes in 20 baseline samples

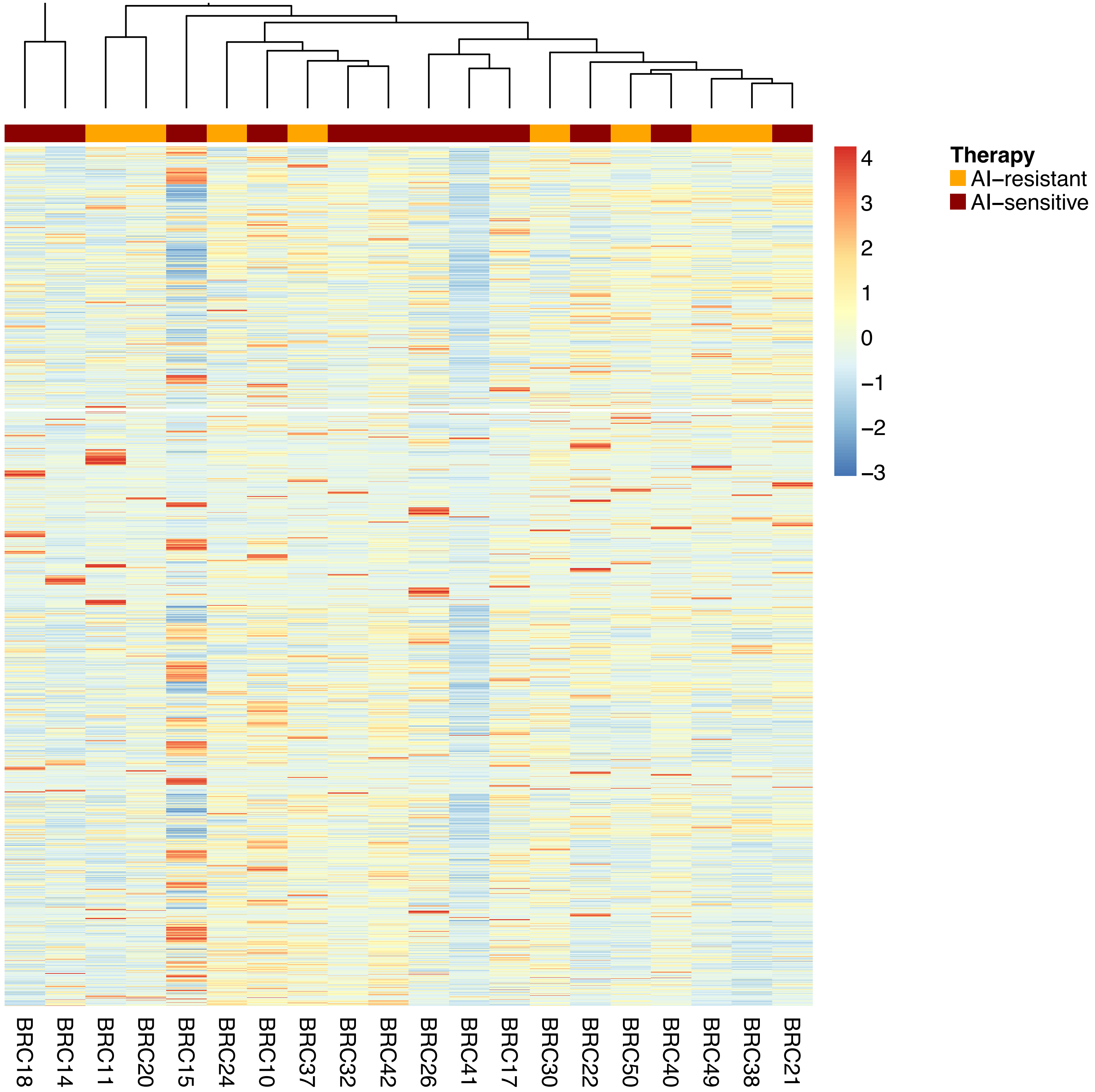

Therapy
AI−resistant
AI−sensitive
4
3
2
1
0
−1
−2
−3
BRC18
BRC14
BRC11
BRC20
BRC15
BRC24
BRC10
BRC37
BRC32
BRC42
BRC26
BRC41
BRC17
BRC30
BRC22
BRC50
BRC40
BRC49
BRC38
BRC21

Supplementary Figure 4: Expression Clustering of surgical samples. Heatmap display of hierachical clustering of the top 5000 most variably-expressed genes in 18 surgical samples

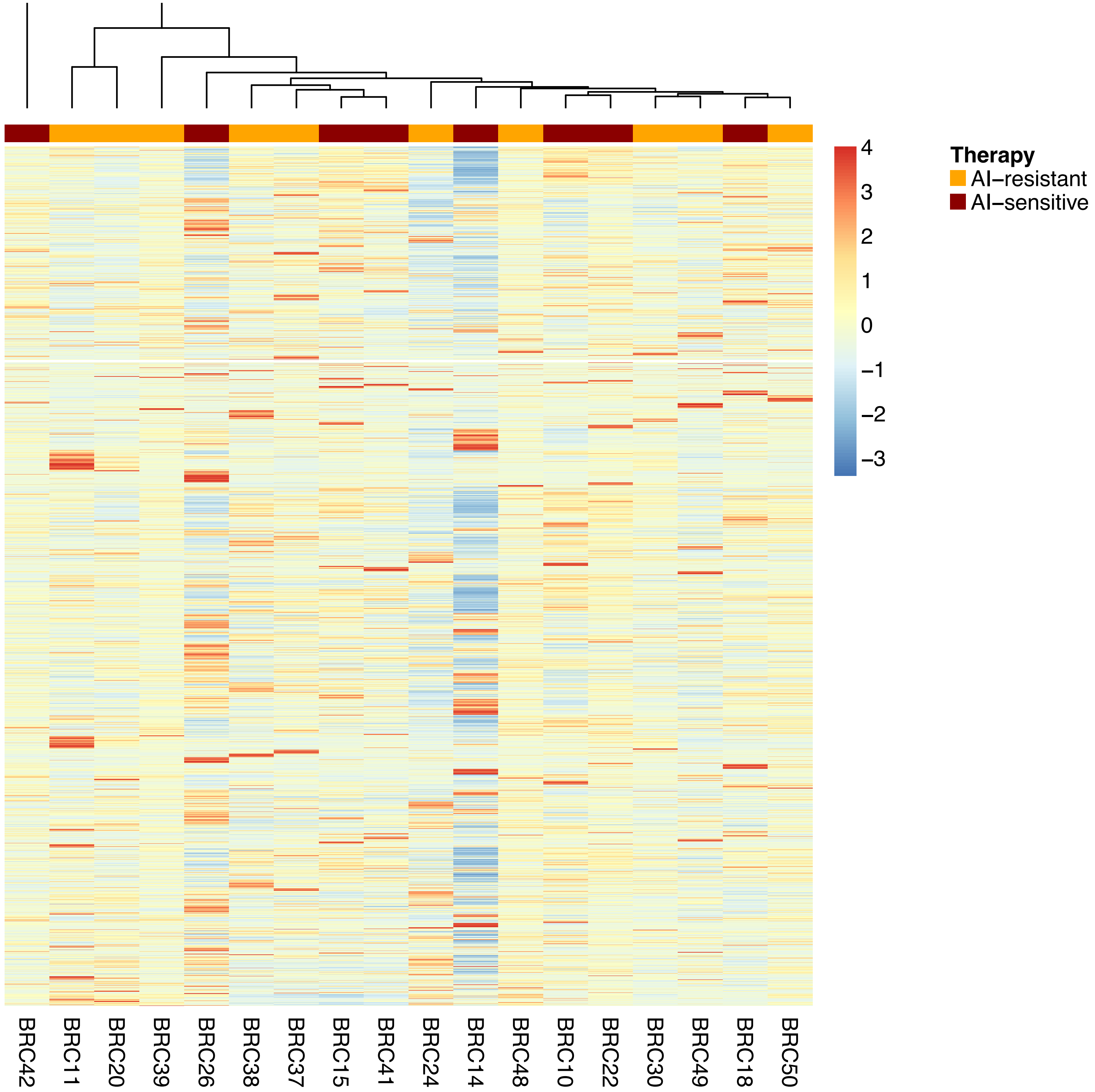

Therapy
AI−resistant
AI−sensitive
4
3
2
1
0
−1
−2
−3
BRC42
BRC11
BRC20
BRC39
BRC26
BRC38
BRC37
BRC15
BRC41
BRC24
BRC14
BRC48
BRC10
BRC22
BRC30
BRC49
BRC18
BRC50

Supplementary Figure 5: Expression Clustering of baseline Luminal A samples. Heatmap display of hierachical clustering of the top 5000 most variably-expressed genes in all baseline LumA samples

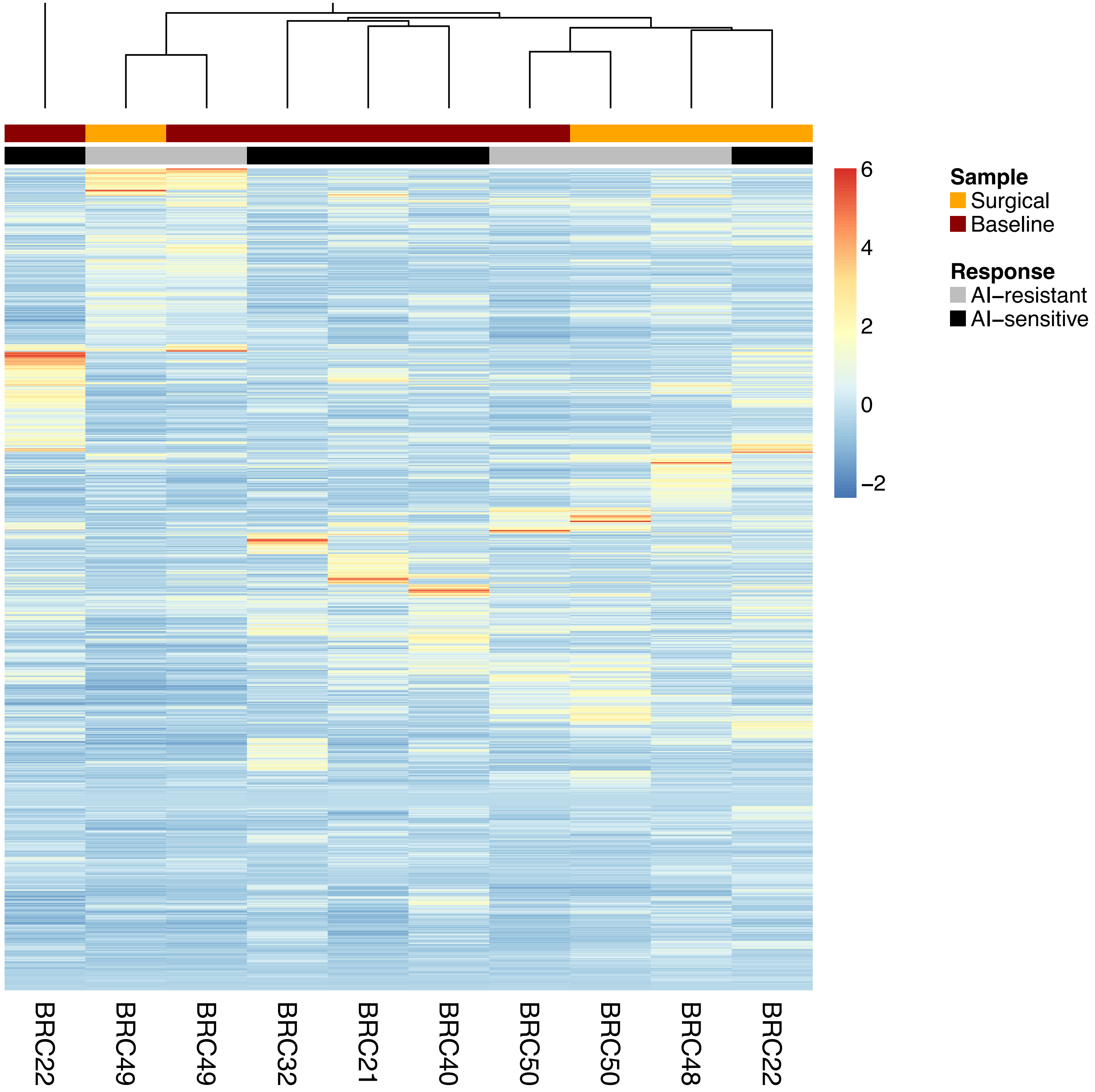

Sample
Surgical
Baseline
Response
AI–resistant
AI–sensitive
6
4
2
0
−2
BRC22
BRC49
BRC49
BRC32
BRC21
BRC40
BRC50
BRC50
BRC48
BRC22

Supplementary Figure 6: Expression Clustering of baseline Luminal B samples. Heatmap display of hierachical clustering of the top 5000 most variably-expressed genes in all baseline LumB samples

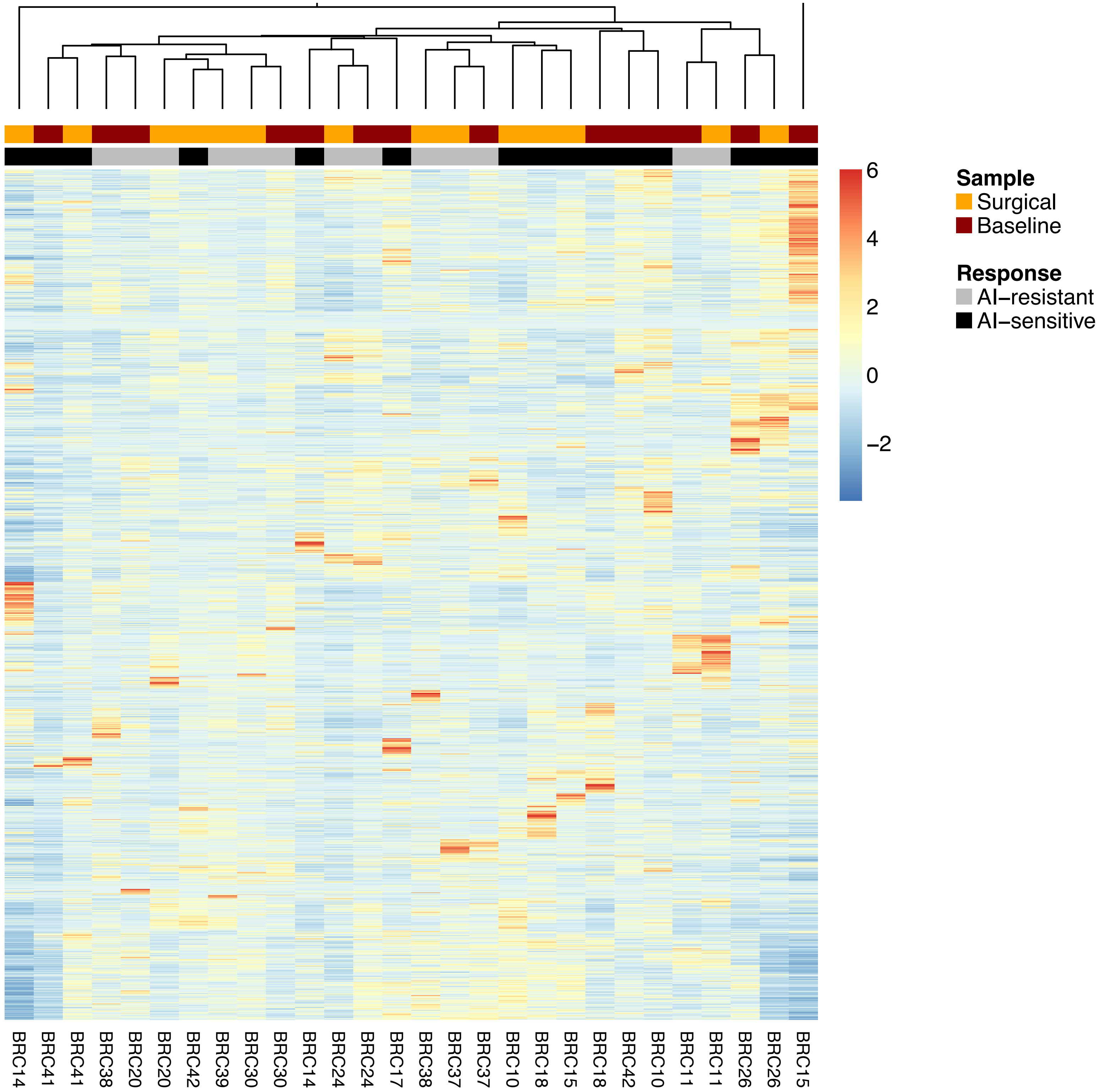

Sample
Surgical
Baseline
Response
AI–resistant
AI–sensitive
6
4
2
0
−2
BRC14
BRC41
BRC41
BRC38
BRC20
BRC20
BRC42
BRC39
BRC30
BRC30
BRC14
BRC24
BRC24
BRC17
BRC38
BRC37
BRC37
BRC10
BRC18
BRC15
BRC18
BRC42
BRC10
BRC11
BRC11
BRC26
BRC26
BRC15

Supplementary Figure 7: Clonality inference for all samples

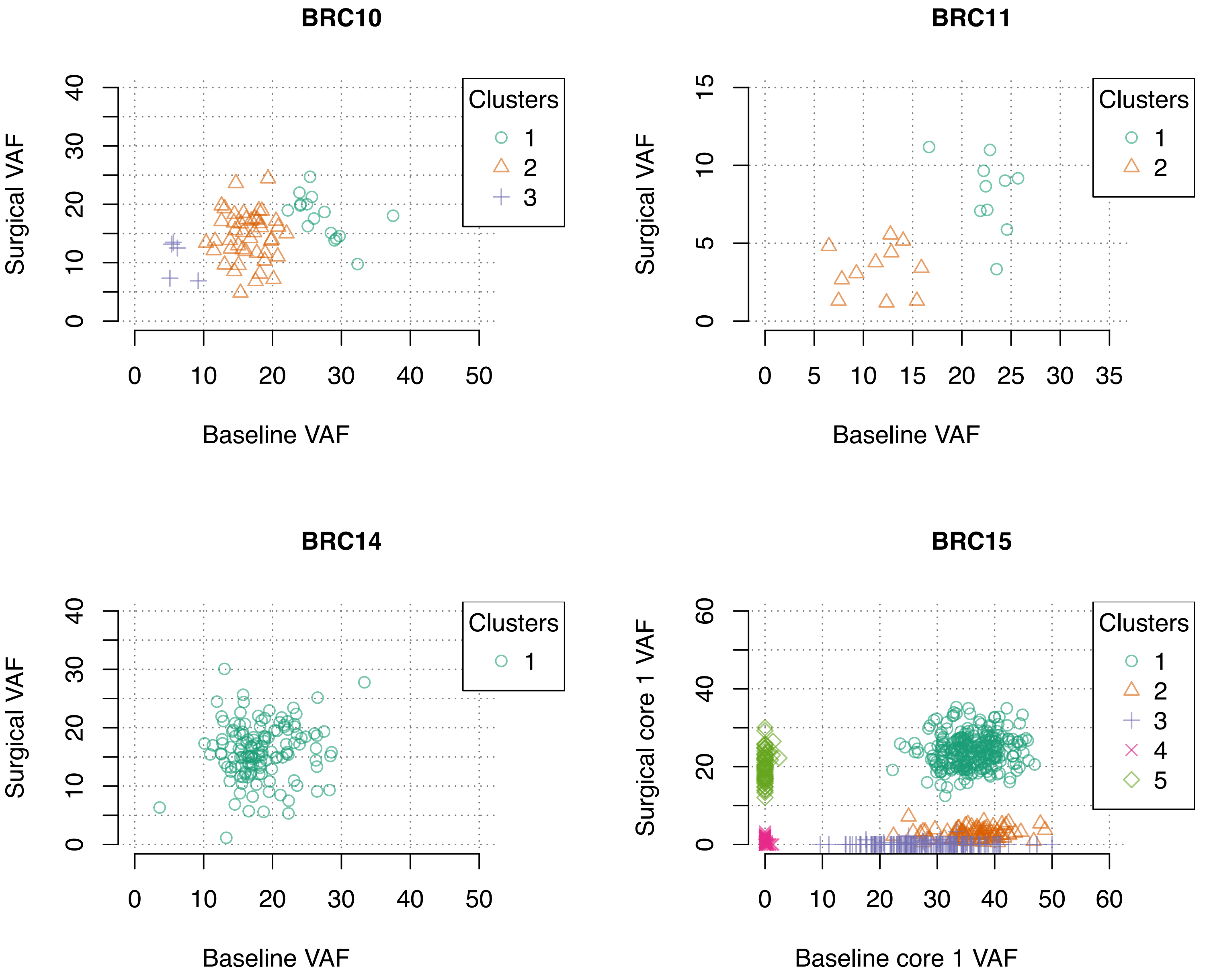
BRC10
Surgical VAF
Baseline VAF
0
10
20
30
40
50
Clusters
1
2
3
BRC11
Surgical VAF
Baseline VAF
0
5
10
15
20
25
30
35
Clusters
1
2
BRC14
Surgical VAF
Baseline VAF
Clusters
1
BRC15
Surgical core 1 VAF
Baseline core 1 VAF
60
Clusters
1
2
3
4
5

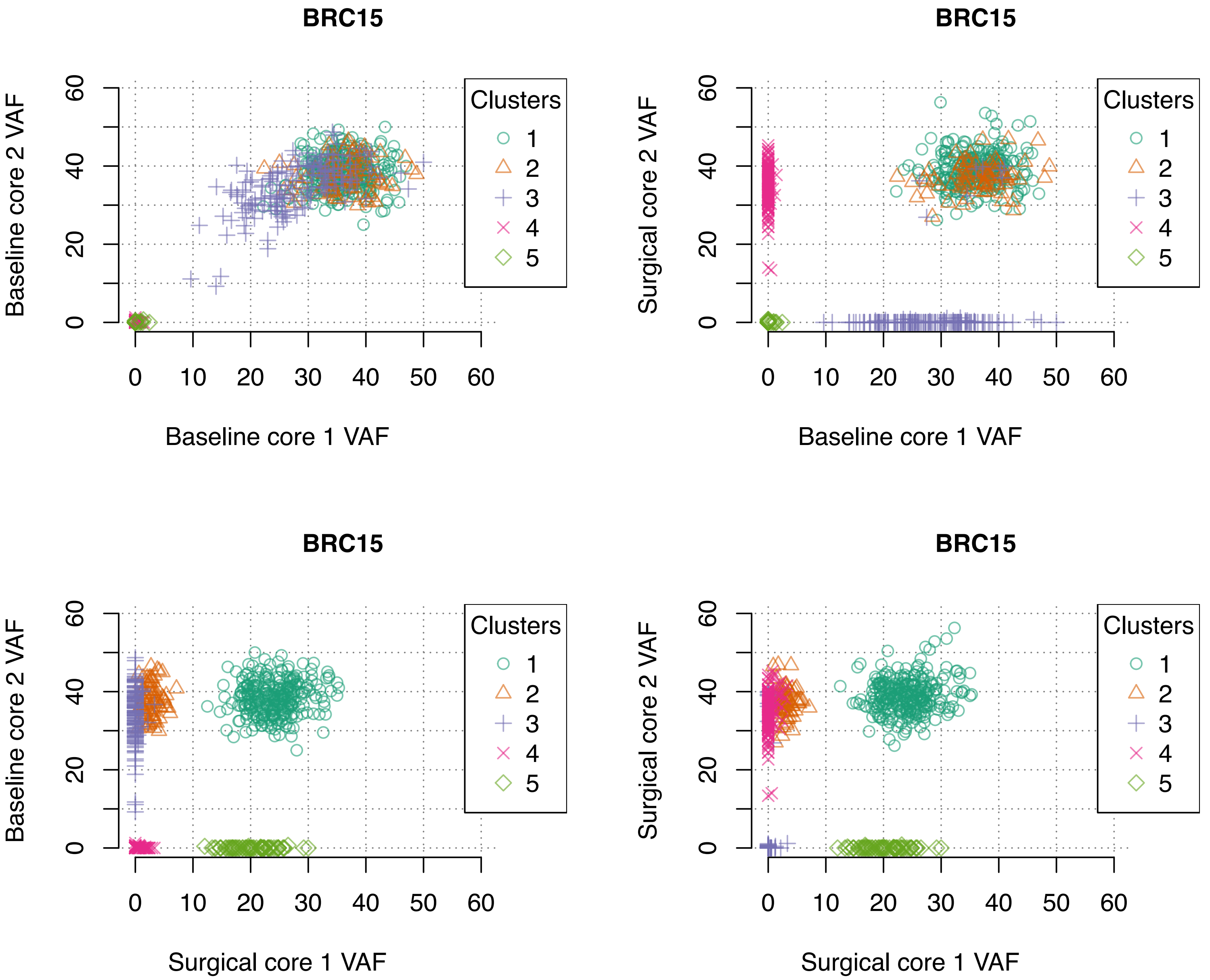
BRC15
Baseline core 2 VAF
Baseline core 1 VAF
Clusters
1
2
3
4
5
0
10
20
30
40
50
60
BRC15
Surgical core 2 VAF
Baseline core 1 VAF
Clusters
1
2
3
4
5
BRC15
Baseline core 2 VAF
Surgical core 1 VAF
Clusters
1
2
3
4
5
BRC15
Surgical core 2 VAF
Surgical core 1 VAF
Clusters
1
2
3
4
5

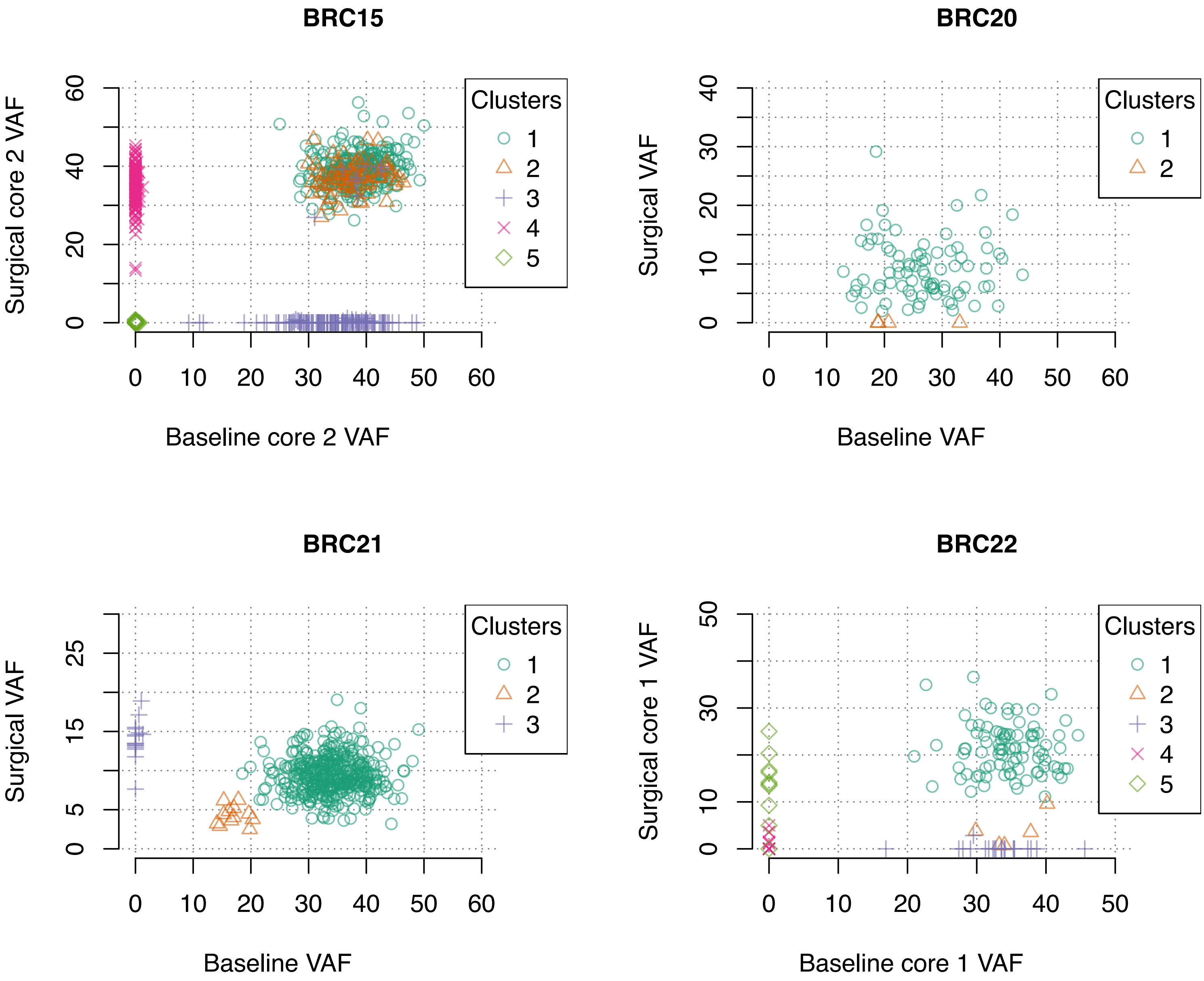

BRC15
Surgical core 2 VAF
Baseline core 2 VAF
Clusters
1
2
3
4
5
0
20
40
60
0
10
20
30
40
50
60
BRC20
Surgical VAF
Baseline VAF
Clusters
1
2
0
10
20
30
40
BRC21
Surgical VAF
Baseline VAF
Clusters
1
2
3
0
5
15
25
BRC22
Surgical core 1 VAF
Baseline core 1 VAF
Clusters
1
2
3
4
5
0
10
30
50

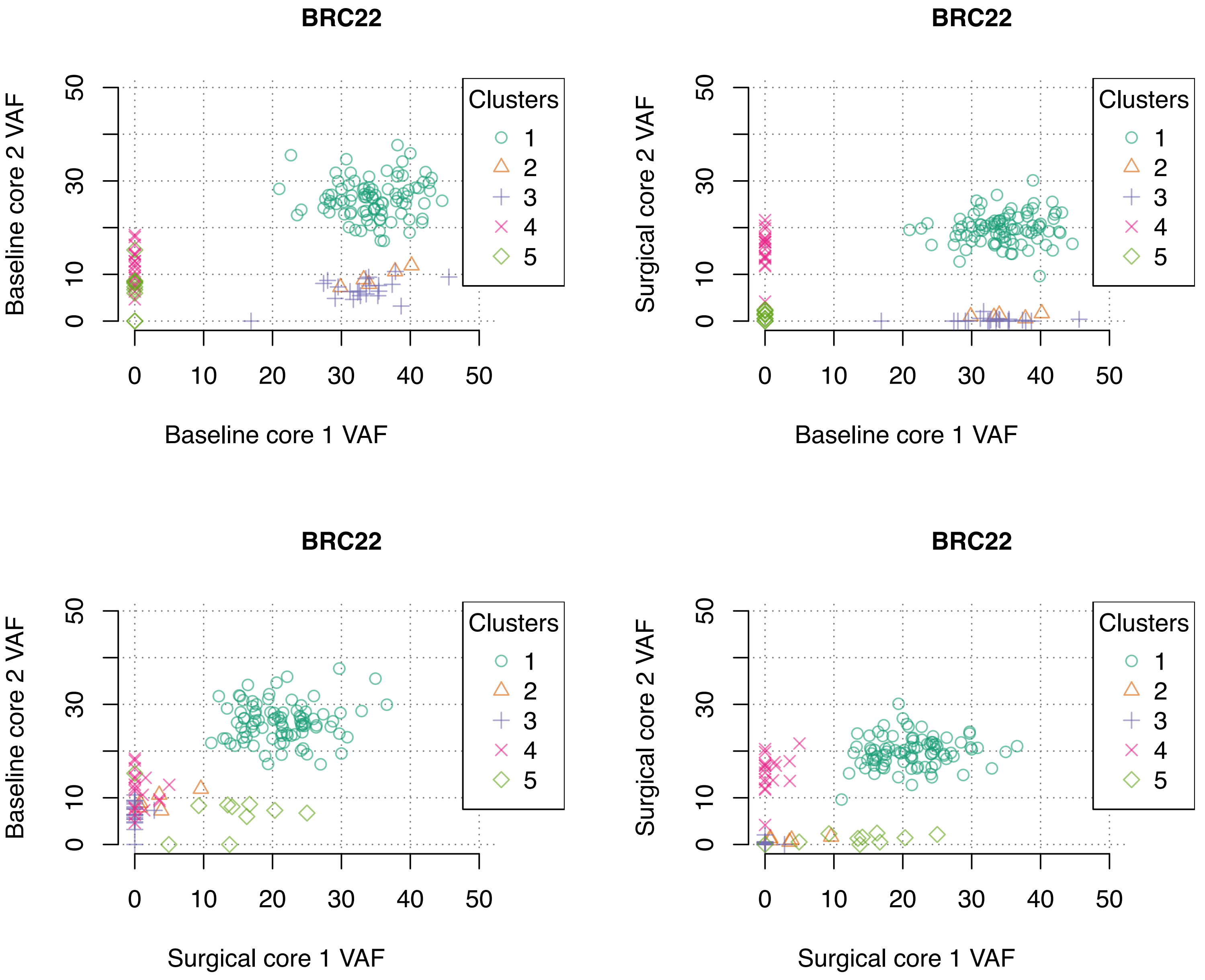

BRC22
Baseline core 2 VAF
Baseline core 1 VAF
Clusters
1
2
3
4
5
0
10
20
30
40
50
BRC22
Surgical core 2 VAF
Baseline core 1 VAF
BRC22
Baseline core 2 VAF
Surgical core 1 VAF
BRC22
Surgical core 2 VAF
Surgical core 1 VAF

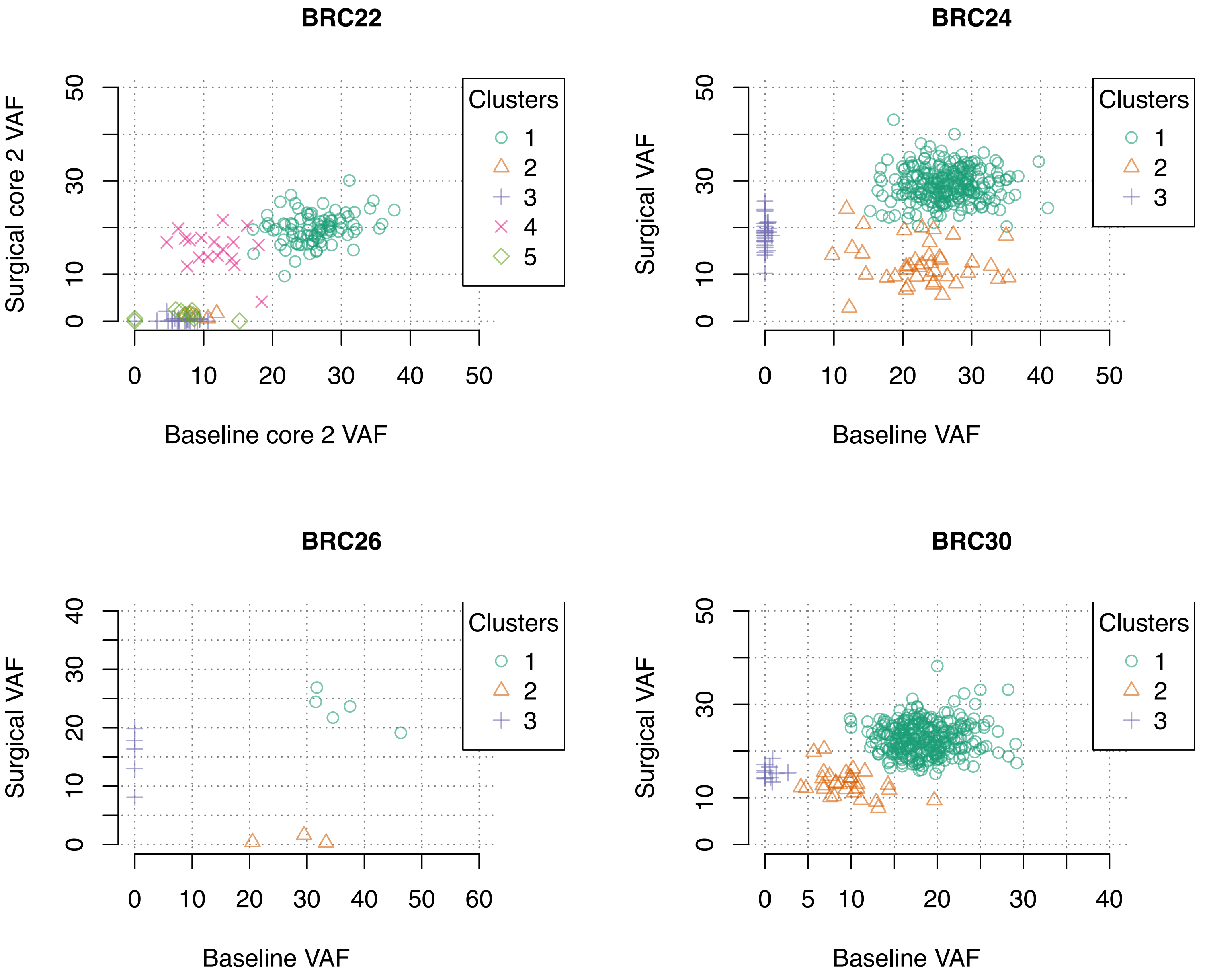
BRC22
Surgical core 2 VAF
Baseline core 2 VAF
Clusters
1
2
3
4
5
BRC24
Surgical VAF
Baseline VAF
Clusters
1
2
3
BRC26
Surgical VAF
Baseline VAF
Clusters
1
2
3
BRC30
Surgical VAF
Baseline VAF
Clusters
1
2
3

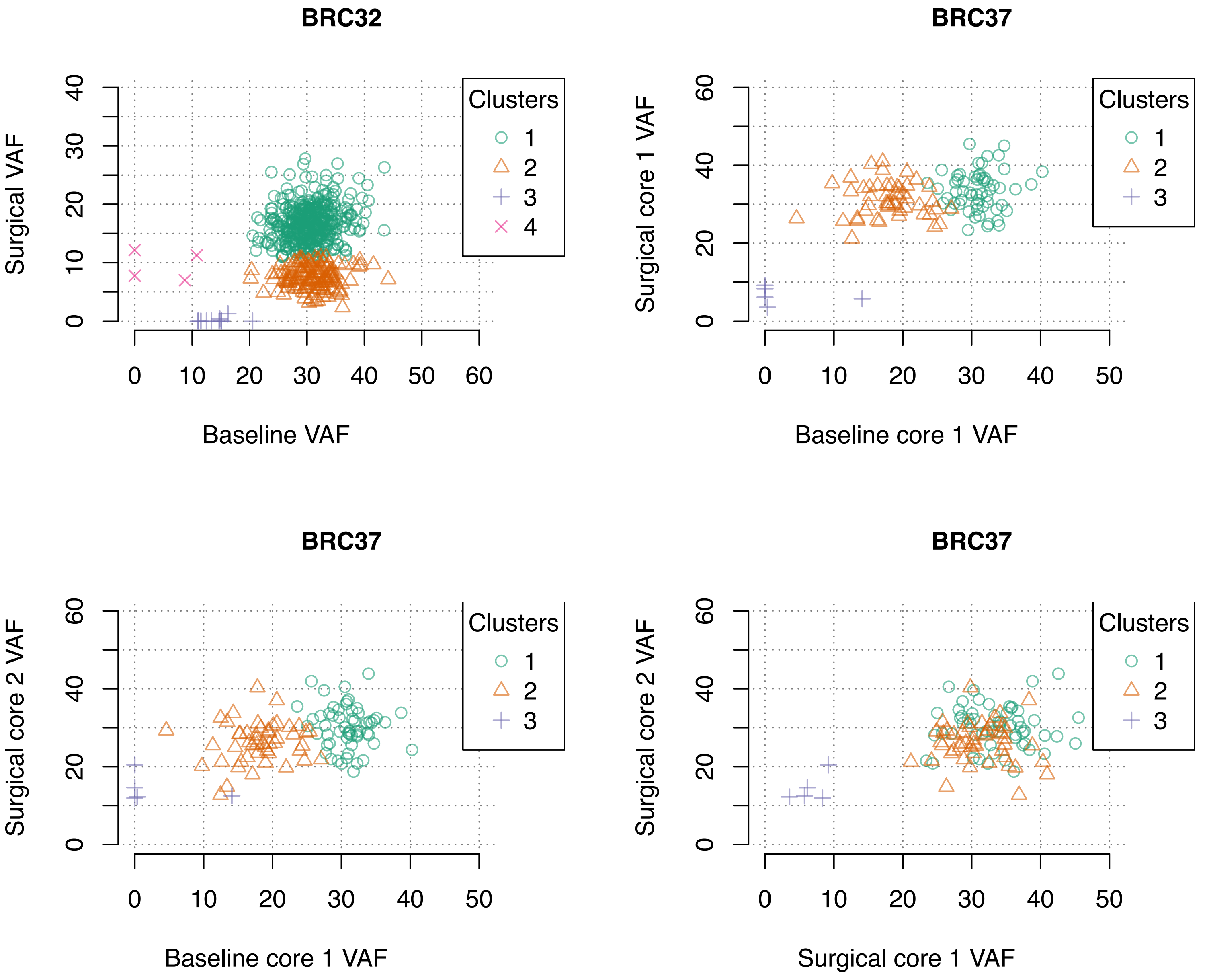

BRC32
Surgical VAF
Baseline VAF
Clusters
1
2
3
4
BRC37
Surgical core 1 VAF
Baseline core 1 VAF
Clusters
1
2
3
BRC37
Surgical core 2 VAF
Baseline core 1 VAF
Clusters
1
2
3
BRC37
Surgical core 2 VAF
Surgical core 1 VAF
Clusters
1
2
3

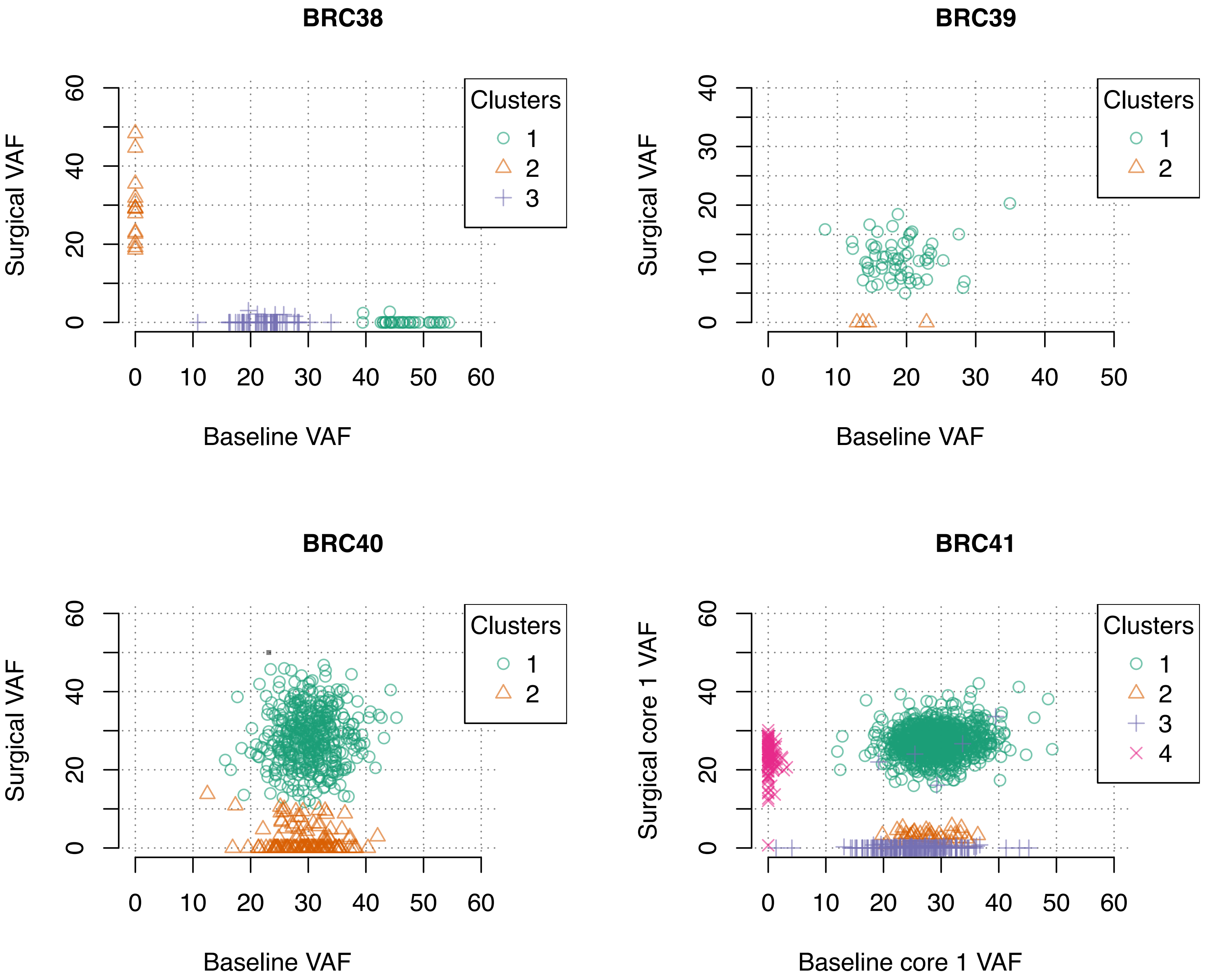

BRC38
Surgical VAF
Baseline VAF
Clusters
1
2
3
0
20
40
60
0
10
20
30
40
50
60
BRC39
Surgical VAF
Baseline VAF
Clusters
1
2
0
10
20
30
40
0
10
20
30
40
50
BRC40
Surgical VAF
Baseline VAF
Clusters
1
2
0
20
40
60
0
10
20
30
40
50
60
BRC41
Surgical core 1 VAF
Baseline core 1 VAF
Clusters
1
2
3
4
0
20
40
60
0
10
20
30
40
50
60

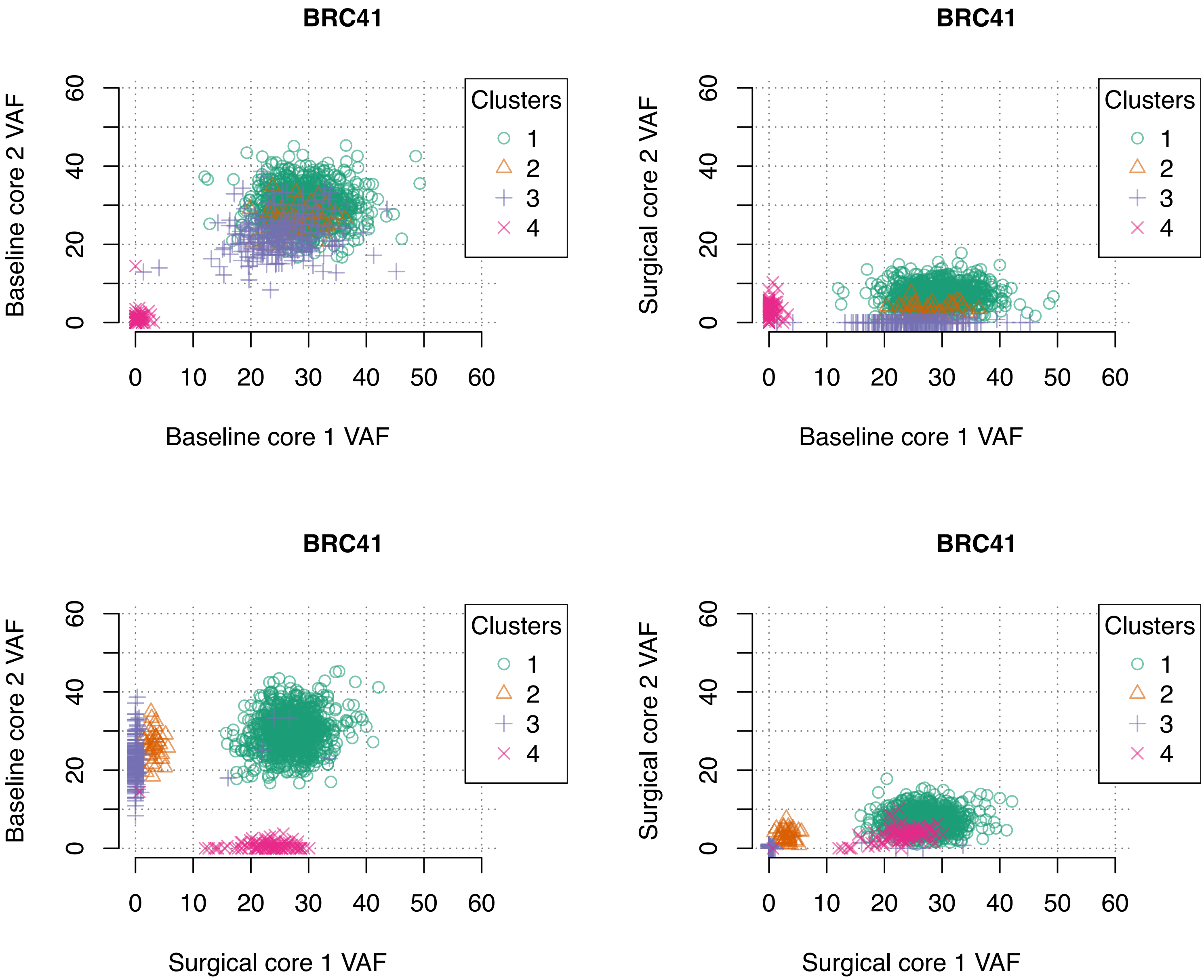
BRC41
Baseline core 2 VAF
Baseline core 1 VAF
Clusters
1
2
3
4
0
10
20
30
40
50
60
BRC41
Surgical core 2 VAF
Baseline core 1 VAF
BRC41
Baseline core 2 VAF
Surgical core 1 VAF
BRC41
Surgical core 2 VAF
Surgical core 1 VAF

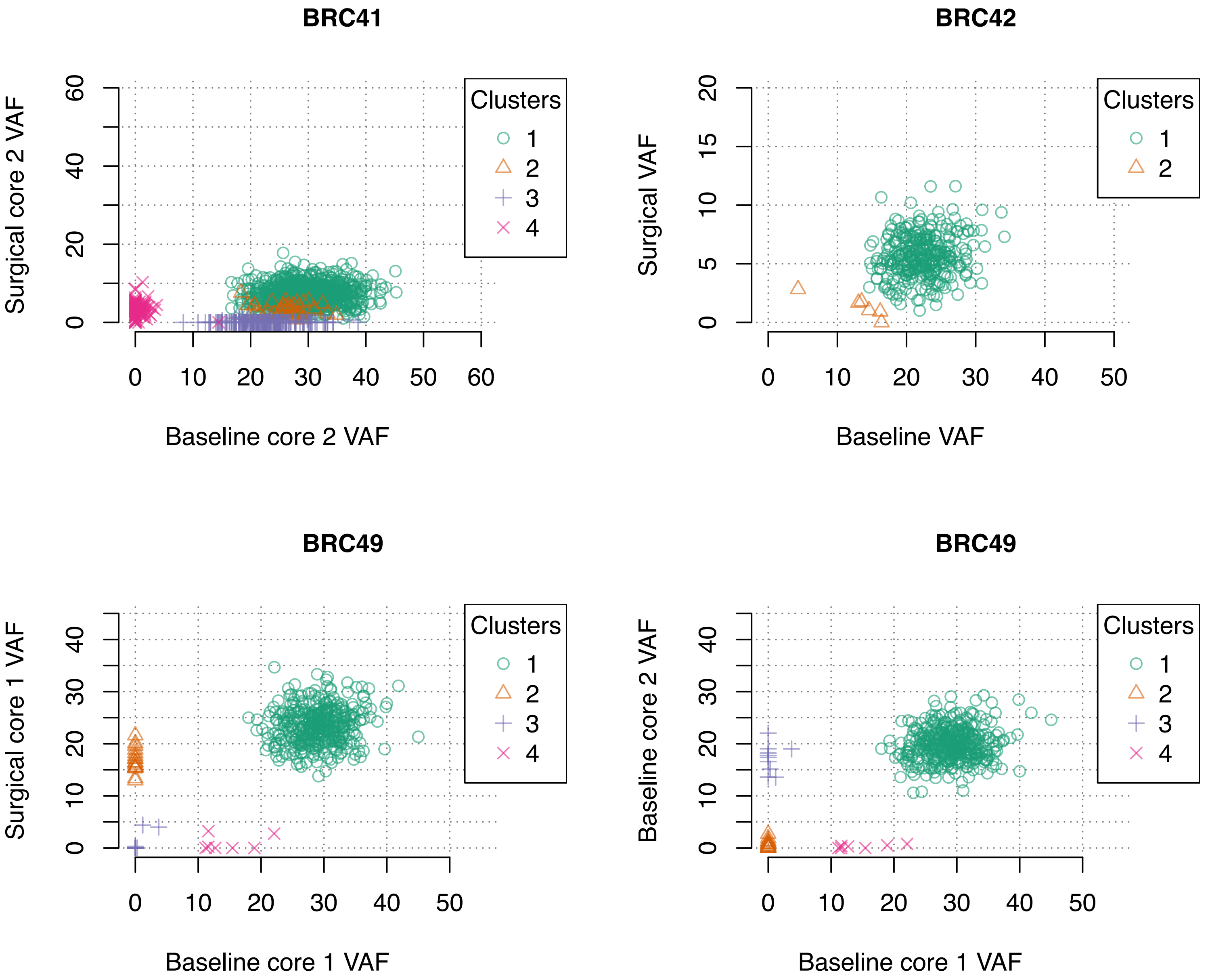

BRC41
Surgical core 2 VAF
Baseline core 2 VAF
0
20
40
60
0
10
20
30
40
50
60
Clusters
1
2
3
4
BRC42
Surgical VAF
Baseline VAF
0
5
10
15
20
0
10
20
30
40
50
Clusters
1
2
BRC49
Surgical core 1 VAF
Baseline core 1 VAF
0
10
20
30
40
0
10
20
30
40
50
Clusters
1
2
3
4
BRC49
Baseline core 2 VAF
Baseline core 1 VAF
0
10
20
30
40
0
10
20
30
40
50
Clusters
1
2
3
4

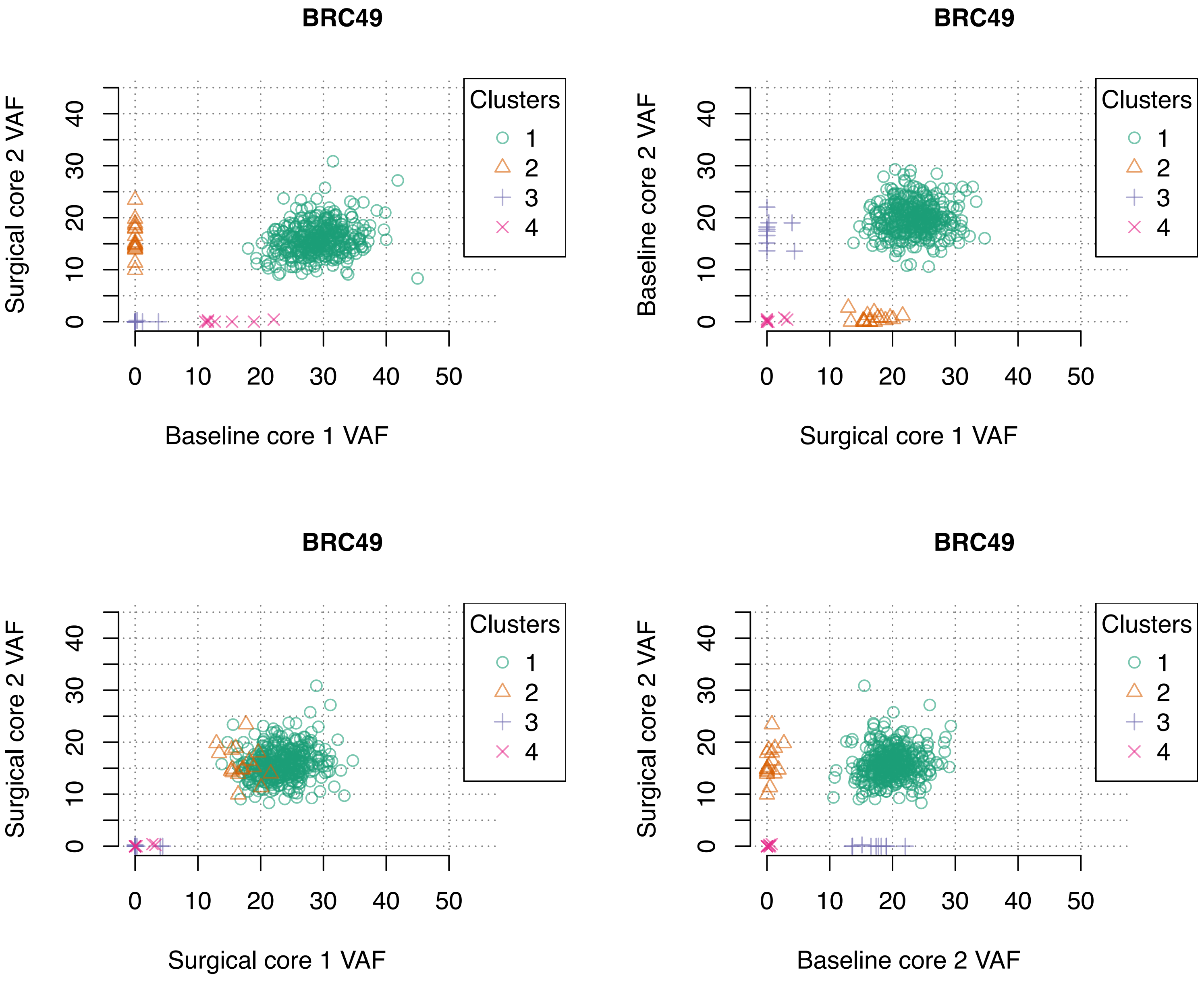
BRC49
Surgical core 2 VAF
Baseline core 1 VAF
Clusters
1
2
3
4
BRC49
Baseline core 2 VAF
Surgical core 1 VAF
Clusters
1
2
3
4
BRC49
Surgical core 2 VAF
Surgical core 1 VAF
Clusters
1
2
3
4
BRC49
Surgical core 2 VAF
Baseline core 2 VAF
Clusters
1
2
3
4

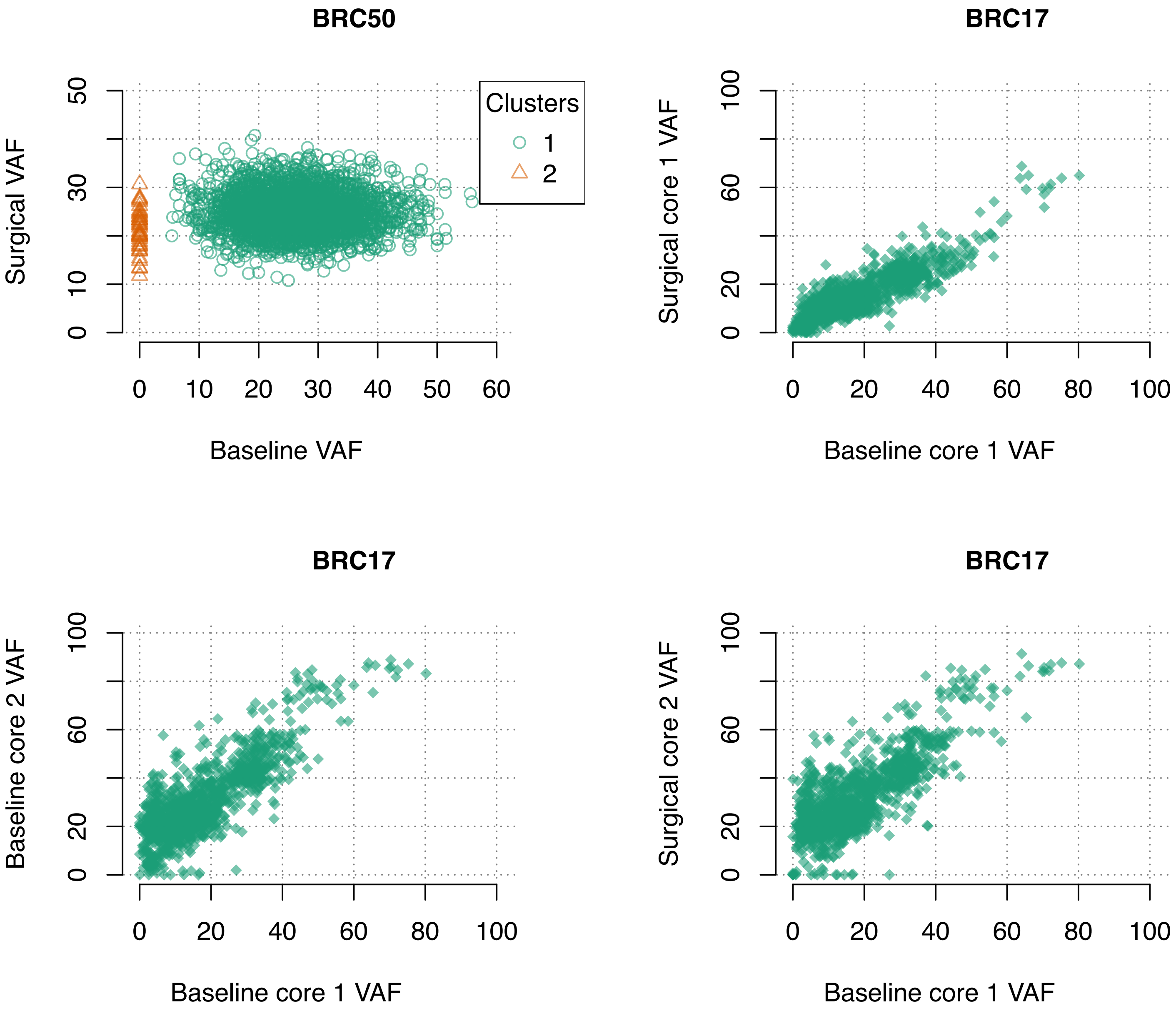

BRC50
Clusters
1
2
Surgical VAF
Baseline VAF
0
10
20
30
40
50
60
BRC17
Surgical core 1 VAF
Baseline core 1 VAF
0
20
40
60
80
100
BRC17
Baseline core 2 VAF
Baseline core 1 VAF
BRC17
Surgical core 2 VAF
Baseline core 1 VAF

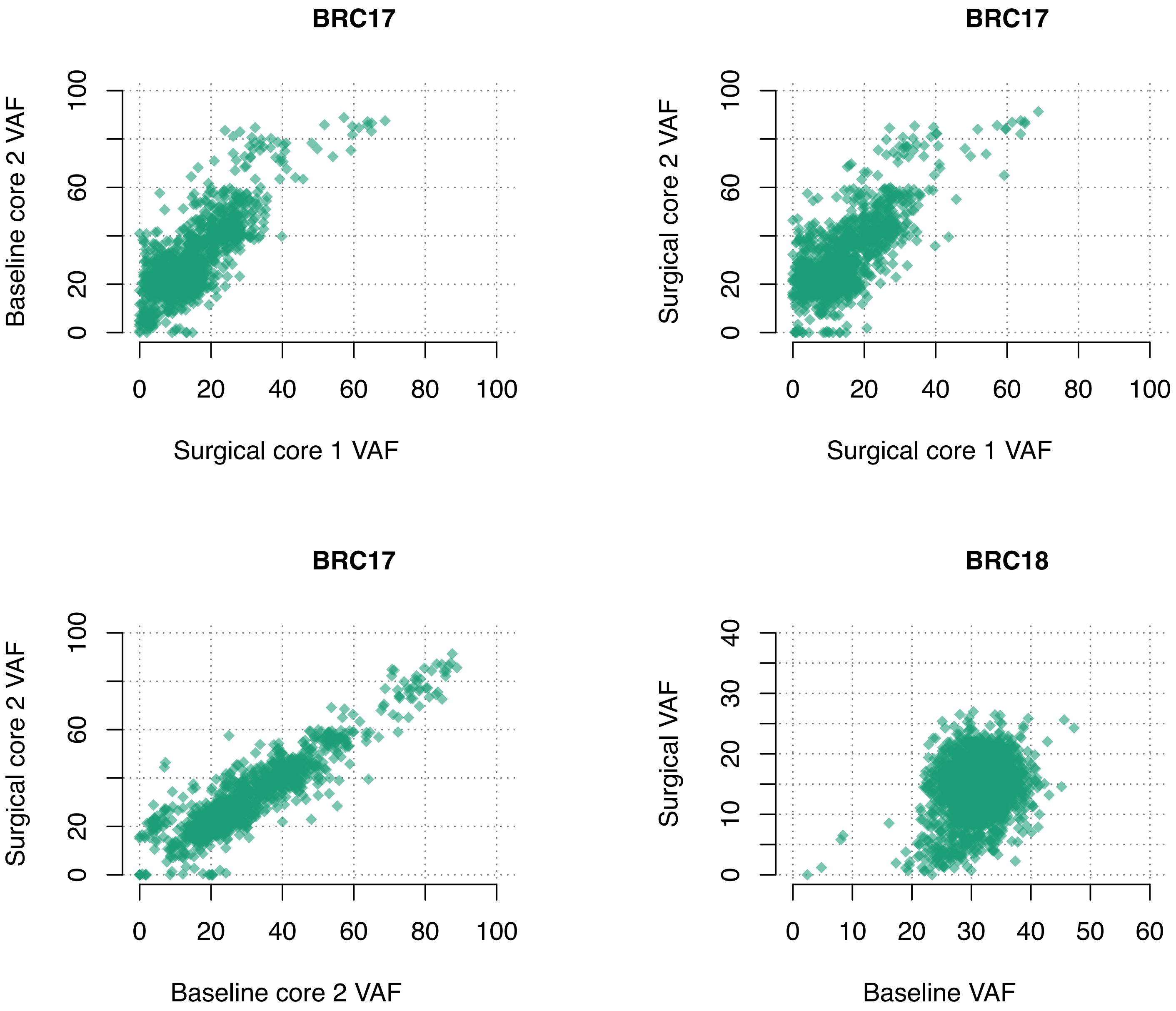

BRC17
Baseline core 2 VAF
Surgical core 1 VAF
0
20
60
100
0
20
40
60
80
100
BRC17
Surgical core 2 VAF
Surgical core 1 VAF
BRC17
Surgical core 2 VAF
Baseline core 2 VAF
BRC18
Surgical VAF
Baseline VAF
0
10
20
30
40
50
60

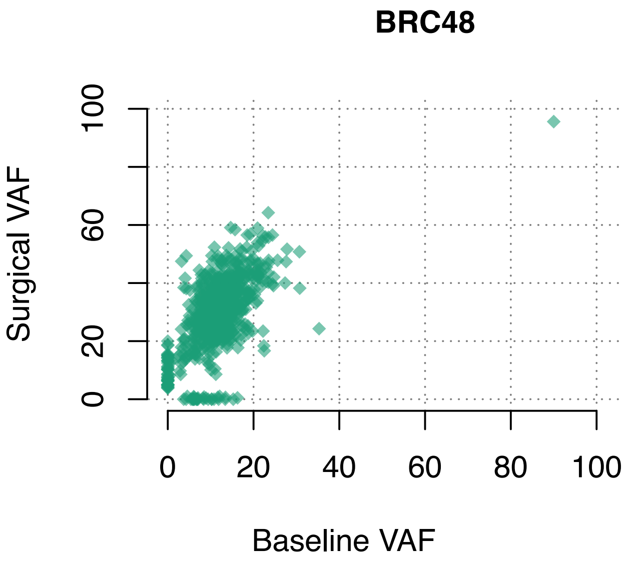
BRC48
Surgical VAF
Baseline VAF
0
20
40
60
80
100

Supplementary Figure 8: VAF changes of mutations in key genes in the extension cohort. No gene had a shift in a positive or negative direction that reached significance in this cohort.

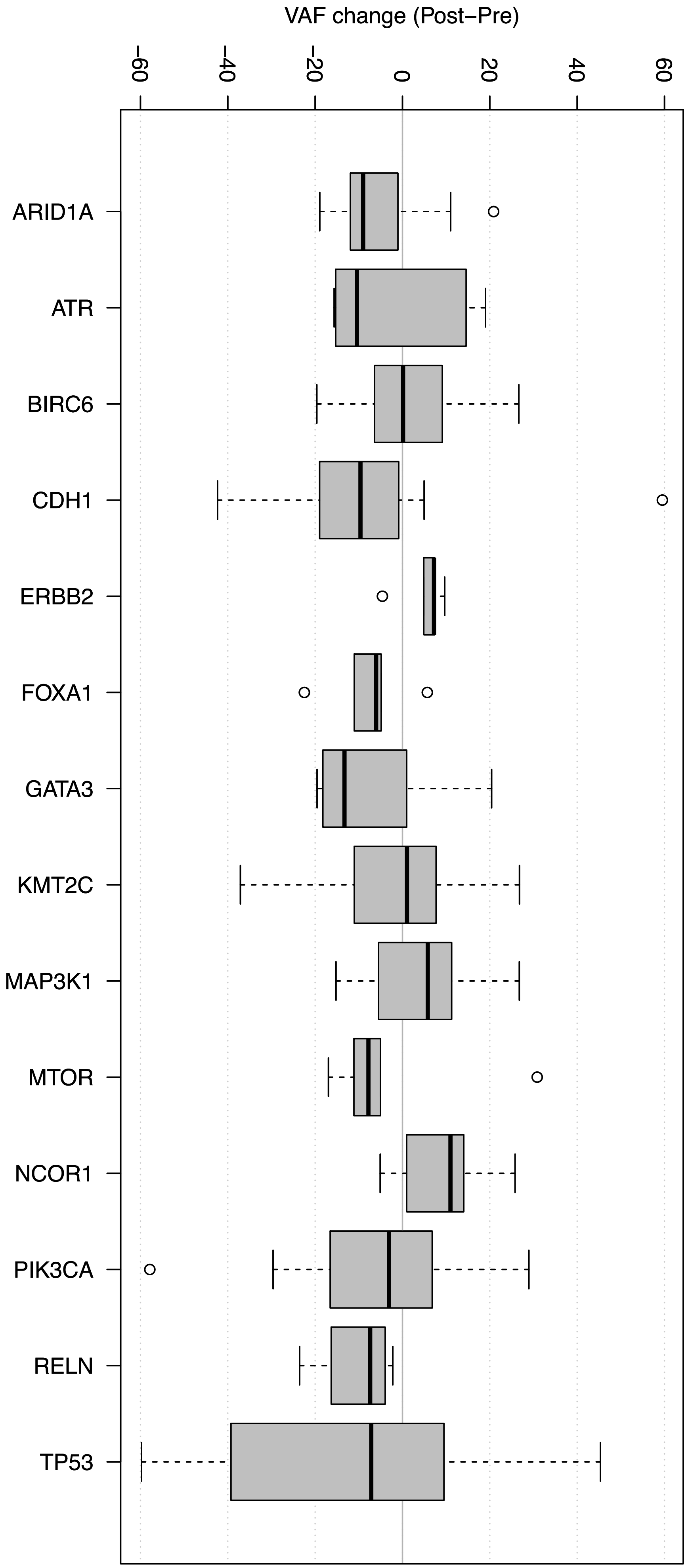

VAF change (Post−Pre)
−60
−40
−20
0
20
40
60
ARID1A
ATR
BIRC6
CDH1
ERBB2
FOXA1
GATA3
KMT2C
MAP3K1
MTOR
NCOR1
PIK3CA
RELN
TP53

Supplementary Notes

Supplementary Note 1: Integrated Analysis of DNA and RNA

30.6% of all coding mutations were expressed at detectable levels in the RNA data. In an attempt to identify specific drivers of response and resistance, baseline- and surgical-specific variants were identified and further subset to retain only expressed variants. Key cancer-related mutations found specifically in surgical samples include BCAS3 (p.K533N) and MLLT4 (p.M855L). Mutations observed in the baseline samples but completely absent in the surgical tumors include MSH6 (p.K1140*), BRAF (p.K601E), FOXA1 (p.R265H), as well as a splice-site mutation in CDH1. In BRC21, we also observe a large reduction in the VAF of a GATA3 splice-site mutation, declining from nearly every cell in the baseline-detected tumor to only about 19% of them. No changes of this type were detected in ESR1 or in related pathway genes. Sixty-two expressed coding mutations were specific to the surgical samples

Supplementary Note 2: Identification of Gene Fusions

With the INTEGRATE algorithm, we utilized both DNA and RNA sequence data to identify putative expressed gene fusion transcripts. Out of 38 samples with RNA sequence data, we detected a total of 612 fusion candidates with supporting reads/evidence in both RNA and DNA sequence data (Supplementary Data 6). Out of these candidates, 147 are “tier1”, having higher confidence due to identification of both encompassing and spanning reads. Cancer-related genes involved in fusions include TP53 (BRC30), JAK1 (BRC41, BRC15, and BRC20), and FLT3 (BRC26 post treatment).

Supplementary Note 3: Transcriptome analysis

RNA sequencing was performed on both pre and post-treatment tumors, allowing us to do detailed differential expression analysis. We identified 44 genes that were differentially expressed between the AI-sensitive and AI-resistant tumors (Supplementary Data 8): 24 genes with higher expression in AI-resistant samples and 20 genes with higher expression in AI-sensitive tumors.

Unsupervised hierarchical clustering of FPKM values grouped 16 of the 32 baseline and surgical samples from the same patient together (Supplementary Figure 4), indicating that in those cases, inter-tumor expression variability was greater than expression changes induced by therapy. Such clustering failed to find a clear signature that differentiates between AI-sensitive and AI-resistant tumors, whether given inputs of baseline, surgical, or all tumors (Supplementary Figures 5-6), and whether we evaluated all genes or a subset of the most variable genes. Clustering of Luminal A and B tumors independently also did not distinguish between response classes (Supplementary Figures 7-8).

While transcription levels of estrogen-receptor pathway genes (as defined by GO terms or KEGG pathways) were often variable between timepoints, differences in their transcription levels between baseline and surgical samples did not have a clear relationship to the clonal instability of the tumor or the response to therapy.

Supplementary Note 4: Spatial organization of the tumors

All cores were taken according to the study protocol, but the locations would have been essentially random due to two factors: The first is that when dealing with fresh tissue biopsies, the center and periphery of the tumor are only grossly identifiable by visual inspection. The second is that this was a multi-center trial and there were no specific guidelines about biopsy location communicated to the many surgeons through the study protocol. Thus, this study cannot answer questions about the spatial organization of the tumors (nor was it designed to).

Supplementary Note 5: DCIS Samples from BRC15

Three additional samples from the periphery of BRC15 tumor were obtained at surgery, all classified as Ductal Carcinoma in situ (DCIS) by pathology. Each was sequenced with a targeted panel of 83 breast-cancer related genes (Supplementary Data 11). Two of the DCIS samples contained *BIRC6* and *PIK3CA* mutations identical to those observed in the tumor's founder clone, suggesting that they share a genetic origin with the same pre-neoplastic cells that gave rise to the tumor. The third DCIS sample contained the same pair of founder clone mutations, as well as the observed *BRAF* mutation at a VAF of 13.11% (representing about 35% of the tumor cells in that sample). This indicates a shared ancestry between this lesion and the cells that comprised cluster three in the invasive cancer.
